# Maternal Folate and Vitamin B 12 Concentrations During Pregnancy Influence Neonatal Nutritional Status and Adiposity: Results from the OBESO Cohort

**DOI:** 10.3390/nu17030372

**Published:** 2025-01-21

**Authors:** Isabel González-Ludlow, Ameyalli M. Rodríguez-Cano, Jonatan A. Mendoza-Ortega, Carolina Rodríguez-Hernández, Blanca V. Suárez-Rico, Guadalupe Estrada-Gutierrez, Maricruz Tolentino-Dolores, Sandra B. Parra-Hernández, Maribel Sánchez-Martínez, Sandra Acevedo-Gallegos, Otilia Perichart-Perera

**Affiliations:** 1 Nutrition and Bioprogramming Coordination, National Institute of Perinatology, Mexico City 11000, Mexico; isaglezludlow@icloud.com (I.G.-L.); rocameyalli@gmail.com (A.M.R.-C.); carolina.rdz94@gmail.com (C.R.-H.); cruz_tolentino@yahoo.com.mx (M.T.-D.); 2 Department of Immunobiochemistry, National Institute of Perinatology, Mexico City 11000, Mexico; jonatan.mdz93@gmail.com (J.A.M.-O.); gpestrad@gmail.com (G.E.-G.); rebe1602@hotmail.com (S.B.P.-H.); maribel71sm@yahoo.com.mx (M.S.-M.); 3 Community Interventions Research Branch, National Institute of Perinatology, Mexico City 11000, Mexico; blancasuarezrico@gmail.com; 4 Department of Maternal-Fetal Medicine, National Institute of Perinatology, Mexico City 11000, Mexico; dracevedo_sandra@yahoo.com.mx

**Keywords:** B vitamins o B-complex vitamins, gestation, methyl donor, newborn size, newborn adiposity

## Abstract

Background/Objectives: Folate and B12, among other B vitamins, are methyl donors and contribute to multiple DNA methylation processes. Maternal deficiency of these nutrients may be associated with impaired fetal growth, affecting the nutritional status and adiposity of the newborn. This study aimed to describe maternal folate and B12 status throughout pregnancy and evaluate its association with neonatal nutritional status. Methods: We studied 90 healthy pregnant women and their babies from the prospective OBESO cohort (Mexico City). Serum folate and B12 concentrations were measured (ELISA) in the first and third trimesters of pregnancy. Deficiency was considered if serum folate was <4 ng/mL, red blood cell folate (RBC) < 151 ng/mL, active B12 < 40 pmol/L, and total B12 < 203 pg/mL). Maternal supplementation of these nutrients was recorded. Newborn assessment (24–72 h) included weight (BW), length (L), waist circumference (WC), and fat mass percentage (%FM; air-displacement plethysmography). Newborn nutritional status indexes were computed and interpreted (BMI/age and length/age) (term-WHO, preterm-Intergrowth). Mean differences, correlations, and multiple linear and logistic regressions were performed (SPSS v. 29). Results: One-third of women had total vitamin B12 deficiency at the end of pregnancy; no folate deficiency was observed. High doses for both folic acid and B12 supplementation were identified in the third trimester (2057.04 ± 2100.74 μg/d and 7.35 ± 4.56 μg/d). Higher first- and third-trimester maternal active B12 concentrations predicted higher WC and reduced the risk of LBW. Higher first-trimester Thcy levels increased the risk of stunting. Higher third-trimester total B12 and folate concentrations predicted higher WC; the latter was associated with higher FM% at birth. Conclusions: Maternal folate, B12, and Thcy levels influence newborn nutritional status alterations, including adiposity markers. It is vital to guarantee an optimal and balanced maternal B-complex status throughout pregnancy.

## 1. Introduction

Maternal nutrition before, during, and after pregnancy plays a key role in determining the health and well-being of the offspring. In the short term, poor maternal nutrition is associated with a higher risk of adverse perinatal outcomes, including gestational diabetes, preterm birth, intrauterine fetal growth restriction, altered size at birth, and neonatal death [1,2,3,4,5]. In the long term, nutrition is an essential piece of fetal metabolic programming, which may alter fetal genome expression, leading to permanent structural or physiological alterations and setting adaptive responses that increase the risk of chronic diseases and neurodevelopment alterations later in life [5].

Globally, the prevalence of the term small for gestational age (SGA) has been estimated to be 16.3%, according to a recent review of 158 countries [6]. Size at birth is associated with multiple birth outcomes and long-term health. A recent umbrella review showed that intrauterine fetal growth impairment (SGA) was associated with metabolic and growth alterations, undernutrition, and poor cognitive development. In contrast, fetal rapid growth (Large for gestational age-LGA) was linked to later obesity and cancer [7].

Nutrition during pregnancy is key, considering this stage’s high cellular plasticity and accelerated epigenetic changes. A key pathway in epigenetic gene regulation involves the folate and one-carbon cycle, which generates methyl groups essential for various methylation processes and plays a crucial role in cell division regulation and tissue development. Folate and vitamin B12 are essential nutrients in these cycles. Disruption of any step in these metabolic pathways may result in significant adverse consequences for fetal growth and development [8,9]. Globally, folate deficiency prevalence in women of reproductive age varies from <5% in high-income countries to over 20% in lower-income countries [10], while B12 deficiency prevalence at the beginning of pregnancy has been reported to be around 20% [11]. In 2012, 2% of adult women of reproductive age in Mexico were folate-deficient, and 9% were B12-deficient [10]. Ten years later, no folate deficiency was reported, while 34% had low levels of B12 [11]. Vitamin B12 and folate deficiencies are associated with maternal factors. Obesity increases the risk of both deficiencies. Low dietary folate, lack of supplementation, and the presence of the MTHFR single-nucleotide polymorphism are associated with lower folate blood concentrations. Vegan and plant-based diets and malabsorptive conditions may result in B12 deficiency [12]. Strong evidence exists about the effect of folic acid supplementation on reducing significantly the risk of neural tube defects [13]. Thus, routine supplementation of 400 μg/d is recommended for all pregnant women, with higher doses for those with higher risk [14,15].

Scarce evidence exists about the association between folate and B12 concentrations and offspring nutritional status, particularly with length (and stunting), head circumference, and adiposity markers. Low folate concentrations have been associated with slightly higher offspring body mass index (BMI), higher Thcy levels, and LBW. B12 deficiency appears to be associated with a higher risk of LBW [16]. In children, high maternal folate concentrations with low maternal B12 concentrations have been associated with higher adiposity and insulin resistance at six years of age [17]. This is relevant, considering some guidelines recommend very high doses of folic acid for women at high risk of neural tube defects (NTD) or those with conditions that increase the risk of folate deficiency (e.g., diabetes, obesity, intestinal malabsorption). Excessive folic acid supplementation may result in the accumulation of unmetabolized folic acid (UMFA), competing with 5-MTHF for binding and transportation [1,8,9].

This study aimed to describe maternal methionine and folate cycle markers (folate, B12, and homocysteine) during pregnancy and evaluate their association with a complete newborn nutritional status profile (weight, length, head circumference, body mass index-BMI, stunting, wasting, overweight, and obesity) and adiposity (fat mass percentage, waist circumference). An additional objective was to explore the effect of a maternal high-folate–low vitamin-B12 profile on newborn nutritional status and adiposity.

## 2. Materials and Methods

### 2.1. Subjects

This study derives from the OBESO (Origen bioquímico y epigenético del sobrepeso y la obesidad) cohort, which is being conducted at the National Institute of Perinatology (INPer) in Mexico City (Mexico) since 2017. The study was approved by the Ethics and Research Committees (Registration number: 3300-11402-01-575-17). All procedures were conducted according to the Declaration of Helsinki. Participation was voluntary, and all women signed an informed-consent form agreeing to participate.

Women included in this study were recruited between January 2017 and January 2020. All women were screened for eligibility and invited to participate in the Department of Fetal Medicine. The sample was selected consecutively according to the following inclusion criteria: adult single pregnancy in the first trimester and pregestational body mass index ≥ 18.5. Women were excluded if they had diagnosed diseases (diabetes mellitus, hypertension, uncontrolled thyroid disease, heart, and renal, hepatic, or autoimmune diseases), if congenital structural malformations were detected, or if taking any medications that may affect metabolism (insulin, metformin, corticosteroids). For this analysis, women with pregnancy losses, who started medications, who developed gestational diabetes mellitus or preeclampsia, who did not complete the follow-up, or those without complete vitamin B12, folate, or Thcy analyses in the first and third trimesters of pregnancy were not included in the analysis.

### 2.2. Maternal Assessment

Women were assessed in the Nutrition Clinic in the first (11–13.6 weeks of gestation) and the third (28–34.6 weeks of gestation) trimesters of pregnancy. Demographic and clinical data were obtained. The educational level was considered basic (elementary and middle school), middle (high school/technical education), and higher (university degree). Multiparity was defined as having ≥1 childbirth. Socioeconomic status was classified as low or middle/high. Gestational age at each visit was calculated according to the first-trimester fetal ultrasound. In the first visit, women reported their pregestational weight. The height was measured to the nearest 0.1cm using a digital stadiometer (model 264, SECA, Hamburg, Germany), considering the Frankfort plane for the proper head position. Pregestational BMI (BMI(preg)) was computed, and women were classified as being overweight or having obesity with a BMI(preg) ≥ 25 [18]. Current weight was measured in all visits to the nearest ± 0.1 kg (with light clothing and without shoes), using a calibrated digital scale (model BMB-800, Tanita, Japan). Maternal supplementation (multivitamins, folic acid, and vitamin B12) was prescribed by their obstetrician and was assessed during each visit. Gestational weight gain (GWG) was evaluated in both visits. We classified GWG in the first trimester as excessive if >2.0 kg, adequate if 0–2.0 kg, and insufficient if weight loss was present, according to the Institute of Medicine guidelines [19]. In the third trimester, GWG was classified as excessive, adequate, or insufficient, considering gestational age and the range of weight gain recommended according to the BMI(preg) category.

An 8 h fasting blood sample was collected in each visit. Whole blood (50 μL) was separated and treated with 1% ascorbic acid for posterior red blood cell (RBC) folate analysis. The remaining whole blood was centrifugated at 3500 rpm; three 350 μL aliquots were separated for serum folate, total, and active vitamin B12 measurements. All samples were frozen at <70 °C on the same day of blood collection.

The serum folate, RBC folate, and total and active vitamin B12 concentrations were quantified using the immunoassay technique (ELISA) and the chemiluminescence method. For RBC folate, the measurement considered hematocrit values. For serum and RBC folate, we used Immulite equipment (model 1000, Siemens, Erlangen, Germany). The intraassay variation coefficient was <8%, and the sensitivity was 0.8 ng/mL. For total and active vitamin B12, we used the Architect equipment (model 11000, Abbott Laboratories, Chicago, IL, USA). The accepted variation coefficient for total vitamin B12 was <11% and a <4 pg/mL sensitivity. For active vitamin B12, the accepted variation coefficient was <10%, and the sensitivity was <0.4 pmol/L. When high values were detected, a second analysis was performed in a diluted sample (serum folate, 1:2 and 1:5; RBC, 1:10).

Folate and vitamin B12 deficiencies were considered when serum folate was <4 ng/mL, RBC folate was <151 ng/mL, active B12 was <40 pmol/L, or total B12 was <203 pg/mL, respectively. High serum folate was defined as >20 ng/mL. Elevated Thcy concentration was considered with levels > 15 µmol/L. A high-risk profile (participants with high serum folate and total B12 deficiency) was identified.

### 2.3. Newborn Assessment

Two experienced and trained nutrition professionals performed all anthropometric measurements in newborns, following Lohman’s technique at 24–72 h from birth [20]. Weight was measured to the nearest gram using a Baby/Mommy 1582 pediatric scale (Tanita, Tokyo, Japan). Recumbent length was taken using an infantometer (SECA 207, Hamburg, Germany). Waist circumference (WC) was measured at the level of the umbilicus, after an exhalation, to the nearest millimeter using a W606PM Lufkin tape (Apex Tool Group, Sparks, MD, USA), with the infant in a supine position. Newborn BMI was computed.

Macrosomia and LBW were defined as newborns weighing > 4000 g and <2500 g, respectively. SGA and LGA newborns were classified if weight/age was <10 percentile and >90 percentile, respectively. BMI/age was classified as risk of overweight if the z-score was >1 and as wasting if the z-score was <−2. Length/age was classified as stunting if <−2 z-score. For term newborns (gestational age at birth > 37 weeks), the World Health Organization standards were used [21]; for preterm newborns, the Intergrowth-21st newborn reference data were used [22,23].

Body composition was analyzed in a subgroup of newborns with an air displacement plethysmography device (PEAPOD, COSMED Inc. USA, Concord, CA, USA). Before each measurement, the device was calibrated according to the manufacturer’s protocol. The infant was placed on the Peapod scale for weight measurement without clothes and with a cap on the head. Then, the infant was placed inside the chamber to measure the body volume. Body density was computed, and the infant’s fat mass (FM) was estimated by the equipment’s software using Fomon’s equation. Relative FM (%FM) was recorded.

### 2.4. Statistical Analysis

All descriptive statistics and frequencies were performed. Simple correlations (Spearman) of the continuous study variables and mean differences (Student’s *t*-test or U-Mann–Whitney test, one-way ANOVA or Kruskal–Wallis test) were performed to evaluate associations between folate, B12, Thcy status, and newborn nutritional status. Multivariate linear and logistic regression models were performed for each one-carbon metabolism marker and the change from the first to third trimester; these variables functioned as independent variables. The dependent variables were each neonatal anthropometric measurement as continuous variables (linear models) independently and nutrition status classification as categorical variables (logistic regression models). All models included relevant confounding variables (BMI(preg), gestational weight gain, offspring sex, gestational age at birth, and MVI supplementation) (SPSS v. 28, IBM, Armonk, NY, USA).

## 3. Results

From January 2017 to January 2020, 365 healthy pregnant women were recruited in the cohort. Women who did not complete the prenatal follow-up (*n* = 112), who developed pregnancy complications (preeclampsia *n* = 39, gestational diabetes *n* = 25, or gestational hypertension *n* = 14), took medication (insulin, metformin, steroids; *n* = 10), or who did not have B12 or folate measurements (*n* = 63) during pregnancy were eliminated from the analyses. Of the 102 women who completed the prenatal follow-up, 90 had newborn data collected. We are presenting data from 90 healthy pregnant women and their newborns. Of these newborns, 69 had air displacement plethysmography data available for body composition analysis.

### 3.1. Maternal Analysis

The mean age was 29.6 ± 4.97 years (18 to 40 years). Half of the women had a high level of education, and most were of low socioeconomic status. Little under half of women were multiparous (43.3%; *n* = 39). The mean BMIpreg was 26.07 ± 4.66 kg/m^2^, where the majority of women were classified as having overweight and obesity (Ow/Ob) (Table 1). In the third trimester, gestational weight gain (GWG) was 7.27 ± 4.80 kg, 24.4% showed insufficient weight gain, and 23% (*n* = 21) had excessive gain. By the end of pregnancy, mean folic acid supplementation doses were 2057.04 ± 2100.74 μg/d, and mean B12 supplementation doses were 7.35 ± 4.56 μg/d, where 92.2% (*n* = 83) received folic acid supplementation and 81.1% (*n* = 73) B12 supplementation.

### 3.2. Maternal Folate, Vitamin B12 and Homocysteine Status

Considering total B12 levels, 11.2% (*n* = 10) and 32.2% (*n* = 29) of women were deficient in the first and third trimester, respectively, while according to active B12 levels, 16.7% (*n* = 15) in the first and 14.4% (*n* = 13) in the third trimester were deficient. No folate deficiency (RBC nor serum) was observed during pregnancy. Serum folate concentrations were elevated in 77.8% (*n* = 70) of women in the first trimester and 61.1% (*n* = 55) in the third. A high serum folate/low total B12 pattern was observed in 5.6% of the women (*n* = 5) in the first trimester and in 18.9% (*n* = 17) of women in the third. No elevated concentrations of Thcy were observed during pregnancy.

A tendency towards lower first-trimester active B12 concentrations was found in women with overweight and obesity (*p* = 0.070). Women supplemented with multivitamins and folic acid had higher serum folate concentrations in the third trimester (22.55 ± 9.40 vs. 15.98 ± 7.32 ng/mL; *p* = 0.034) but not in the first trimester. Participants with a higher education level had higher third-trimester serum folate concentrations than those with a middle level (Table 1). No other significant differences were observed between maternal variables and maternal B vitamins and Thcy concentrations during pregnancy (Table 1).

When evaluating changes during pregnancy, total vitamin B12, active B12, serum folate, and Thcy concentrations decreased significantly, whereas RBC folate increased (Table 2).

### 3.3. Neonatal Description

Of all newborns, half were female, and 55.6% (*n* = 50) were born by cesarean section. Mean gestational age at birth was 38.5 ± 2.2 weeks; 8.9% (*n* = 8) of newborns were preterm. The mean newborn anthropometric measurements at birth were as follows: weight, 2887.5 ± 349.06 g; length, 47.22 ± 1.80 cm; WC, 29.57 ± 2.00 cm; and BMI, 12.92 ± 1.04 kg/m^2^. In total, 15.5% of newborns were classified as de SGA, and 13.2% were stunted (Table 3). There were no macrosomic or LGA newborns.

In the first trimester, higher maternal total B12 concentrations were observed in male offspring; in mothers of preterm infants, lower serum folate and higher Thcy was observed compared to those born at term. According to nutritional status, mothers of LBW newborns had lower maternal active B12 levels; those with stunted newborns showed lower serum folate and higher Thcy (Table 3).

In the third trimester, higher maternal concentrations of active B12 were observed in male infants, and higher maternal Thcy concentrations were found in preterm newborns. Considering newborn nutritional status, women with LBW newborns had lower total and active B12 concentrations. SGA newborns also had mothers with lower total vitamin B12 concentrations. Newborns with stunting tended to have higher maternal Thcy concentrations (*p* = 0.080), but this was not statistically significant (Table 4).

Newborns from women with the high serum folate and low total vitamin B12 pattern in the first and third trimesters did not show significant differences in birthweight, length, WC, BMI/age, LBW, stunting, or SGA status, nor according to %FM (*p* > 0.05).

**Figure 1:**
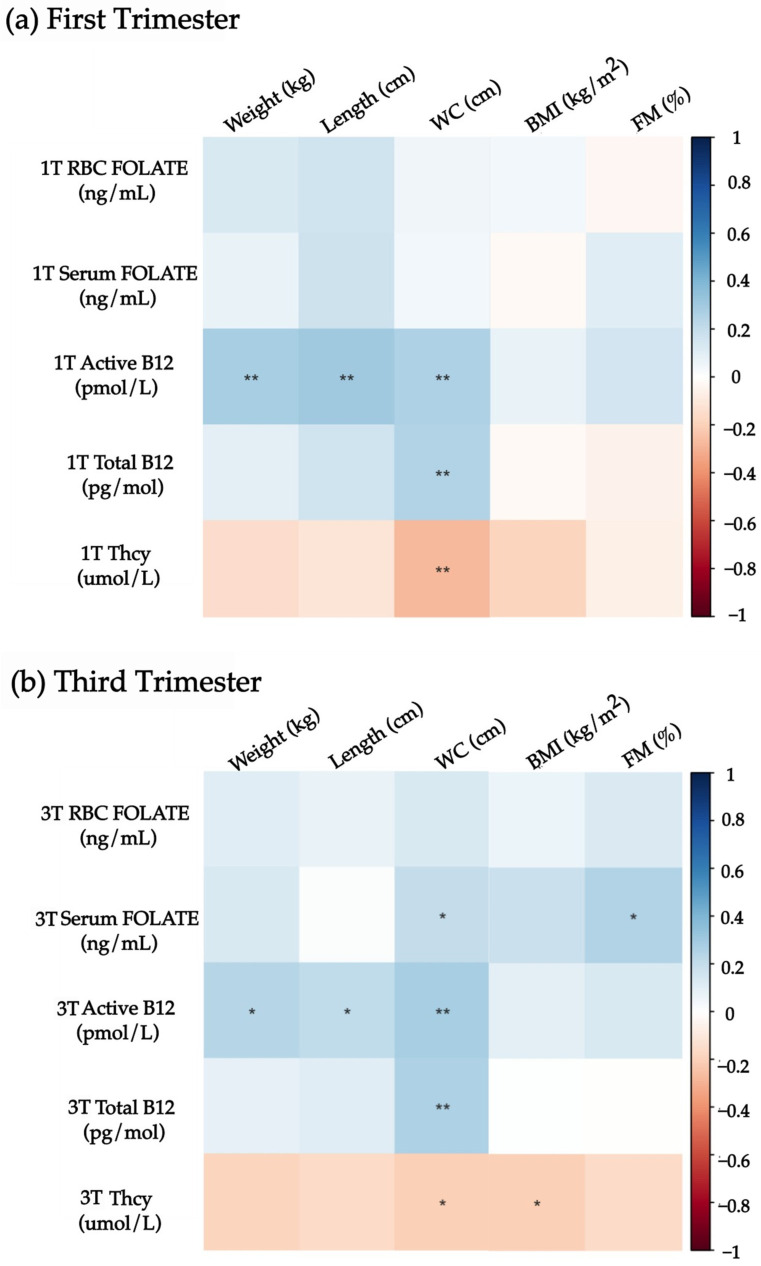
Spearman correlations between maternal folate, vitamin B12, Thcy, and newborn anthropometry and fat mass in the first and third trimester of pregnancy. The right bar shows the “r” value. The blue color represents a positive correlation, and the red color represents an inverse correlation. *p* values < 0.05 are marked as * and *p* < 0.01 as **. 1T: First trimester. RBC: Red blood cells. Thcy: Homocysteine. 3T: Third trimester. BMI: Body mass index. FM: Fat mass. WC: Waist circumference.

Correlations between neonatal nutritional status and maternal B vitamins and Thcy levels are presented in Figure 1. In the first trimester, active B12 was positively correlated with newborn weight, length, and WC, while total B12 was positively correlated with WC. Thcy was negatively correlated to WC at birth. In the third trimester, serum folate was positively correlated to WC at birth; active B12 maintained a positive correlation with weight, length, and WC at birth, and total B12 correlated with WC at birth. Thcy was negatively correlated to neonatal WC and BMI. Maternal serum folate positively correlated with neonatal %FM in those newborns with body composition data.

### 3.4. Associations Between Maternal Folate, Vitamin B12, Homocysteine Status, and Neonatal Nutrition Status

**Figure 2:**
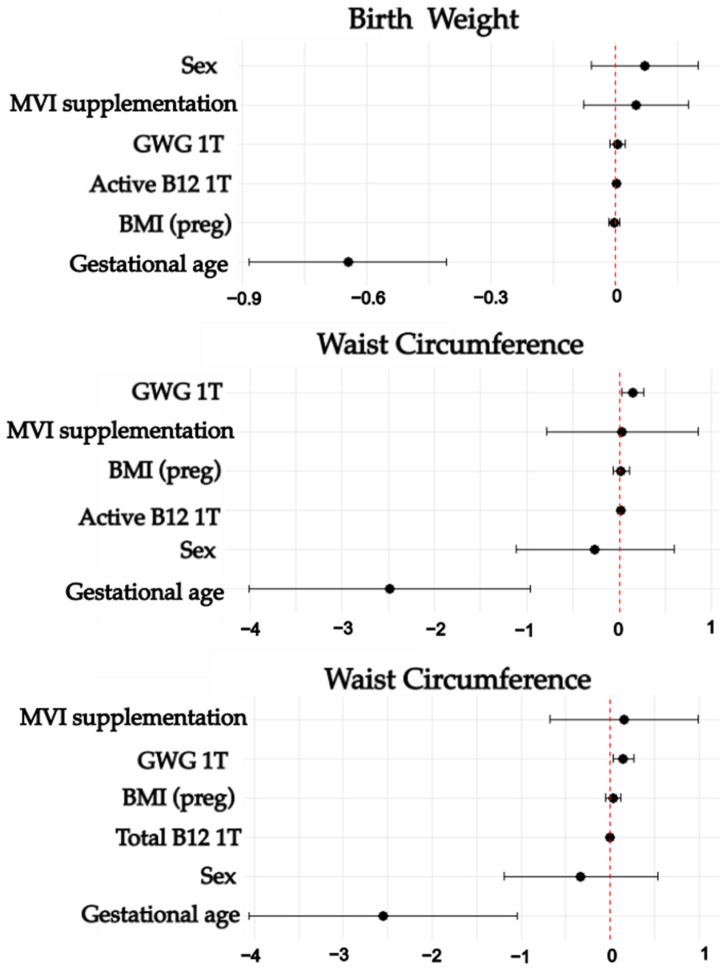
First-trimester maternal B vitamins, homocysteine, and newborn nutritional status. This Figure shows forest plots representing the effect size of the multiple linear regression models. Models were adjusted by multivitamin supplementation, maternal pregestational body mass index, first-trimester gestational weight gain, gestational age at birth, and newborn sex. 1T: First trimester. BMI: Body mass index. MVI: Multivitamin. GWG: Gestational weight gain.

**Figure 3:**
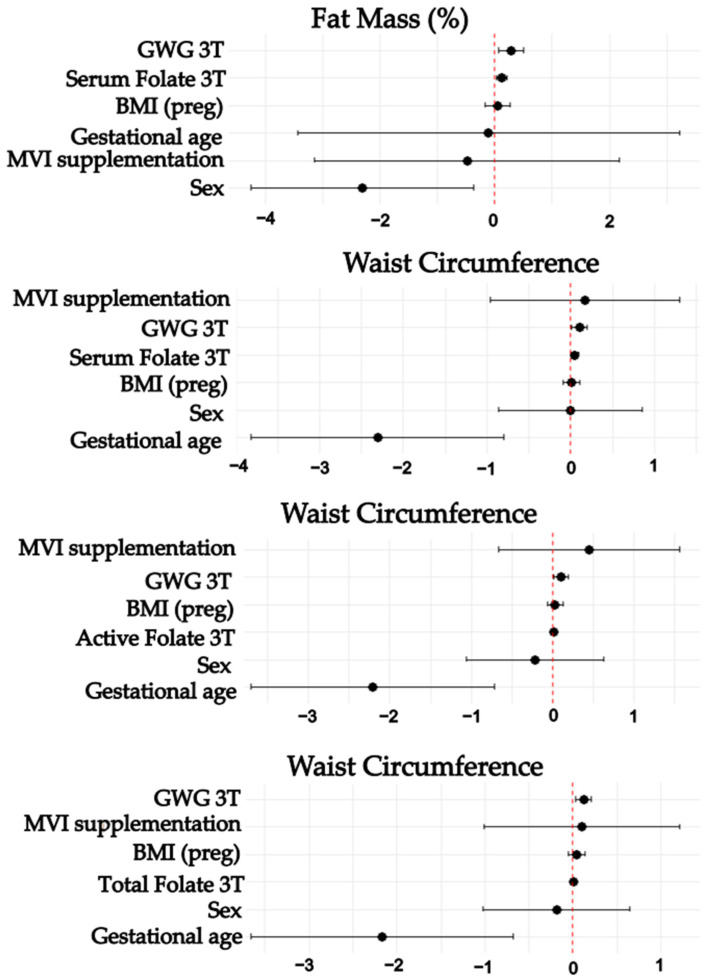
Third trimester maternal B vitamins, homocysteine, and newborn nutritional status. This Figure shows forest plots representing the effect size of the multiple linear regression models. Models were adjusted by multivitamin supplementation, maternal pregestational body mass index, third-trimester gestational weight gain, gestational age at birth, and newborn sex. 3T: Third trimester. BMI: Body mass index. MVI: Multivitamin. GWG: Gestational weight gain.

According to the multivariate analyses, in the first trimester, higher active B12 concentrations predicted a higher birth weight. In addition, first- and third-trimester maternal active and total vitamin B12 concentrations predicted greater WC at birth. In the third trimester, higher folate concentrations predicted a higher WC at birth, while higher Thcy concentrations showed a trend towards significance in predicting LBW (*p* = 0.06). For the FM group, third-trimester folate concentrations predicted a higher %FM. All models included maternal BMI(preg), maternal MVI supplementation, first- or third-trimester GWG (kg), gestational age at birth, and newborn sex as covariables (Table 5 and Table 6; Figure 2 and Figure 3).

Multiple logistic regressions showed that higher concentrations of active vitamin B12 in the first and third trimesters of pregnancy were associated with a reduced risk of LBW. Higher first-trimester Thcy concentrations were associated with a higher risk of stunting (Table 7).

No significant associations were observed when the models (linear and logistic regressions) were run with the change in folate, B12, and Thcy from the first to the third trimester.

## 4. Discussion

In this study, maternal B12, folate, and homocysteine concentrations influenced neonatal nutritional status and adiposity markers.

Higher active first-trimester B12 concentrations were associated with higher birth weight. Maternal imbalance of any one-carbon methyl donor may induce epigenetic changes, modifying cellular mechanisms that could lead to fetal growth, developmental alterations, and metabolic programming. It is known that B12 deficiency during pregnancy results in a limited intrauterine supply and affects fetal storage [24]. Fetal uptake depends entirely on maternal levels. Low maternal levels of B12 have been associated with lower birth weight, impaired cognitive and neurological functions (short- and long-term), and preterm birth [16,25,26,27,28]. These outcomes relate to this vitamin’s functions in the one-carbon metabolism and nucleic acid synthesis, DNA methylation, fetal cellular growth, and tissue differentiation.

In our study, higher active and total B12 and serum folate were associated with higher WC. Nevertheless, evidence regarding the effects of maternal B12 and anthropometric measurements at birth remains inconsistent, as some studies have found inverse or no association at birth [29,30,31,32]. According to the Generation R Study results (*n* = 5890), maternal B12 concentrations were not associated with birthweight or other anthropometric measurement. However, total and active B12 concentrations in the umbilical cord were inversely associated with the offspring’s anthropometric measurements at birth (birthweight, birth length, and birth head circumference) [29]. Deficiencies in both folate and B12 can result in elevated levels of Thcy, which has been linked to LBW and is a significant independent risk factor for vascular complications in pregnancy [33]. When studying iron, folate, and B12 maternal dietary intake in the ROLO longitudinal birth cohort study, there was no association between the intake of these nutrients and fetal abdominal circumference growth trajectory (latent class trajectory mixture models) [34]. The study by Yajnik et al. found that lower maternal folate concentrations were associated with lower abdominal circumference (*p* = 0.003 and 0.008, respectively) [31], similar to what we observed. They also reported an association between low folate status and lower birthweight, which we did not find. In another study performed in our institution (INPer), lower maternal folate concentrations in adolescent mothers increased the risk of having an SGA newborn [35].

As for maternal folate status and neonatal fat mass, we found that higher serum folate concentrations were associated with a higher %FM at birth. To our knowledge, no studies have reported similar findings at birth, but other studies have reported maternal folate status associations with infant adiposity. Yajnik et al. described that elevated maternal RBC folate levels in the third trimester predicted higher offspring adiposity at six years old [31]. On the other hand, Zhang et al. reported that the lack of early maternal folic acid supplementation was related to a “high-body fat ratio trajectory” at 4–6 years old, where fat mass percentage was measured by bioimpedance a minimum of three times [36].

WC, body fat, and BMI have been shown to influence early adiposity rebound in children, specifically, higher adiposity before 72 months of age [36]. In this study, higher levels of B12 and folate predicted a higher WC and FM%, respectively. WC may predict early metabolic risk [37]. However, more studies are needed to elucidate the ideal FM% at these early stages.

We did not observe folate deficiency (RBC or serum) or high Thcy in this group of pregnant women. Still, a high proportion of them (77.8%) had excessive serum folate levels in the first trimester. Even though the proportion of women with high values decreased by the third trimester, it was still represented by more than 50%. This may be partly explained by the high doses of folic acid prescribed to this group of pregnant women since early pregnancy (Median: 900.0 μg/d; IQR: 400.0–1666.0 μg/d). High folic acid doses have been associated with higher serum folate levels but not RBC (similar to our results), denoting tissue saturation [38]. However, RBC folate was the only marker that increased significantly during pregnancy, compared to the other B vitamins, which decreased significantly. This probably reflects how the body compensates to preserve stores for the postnatal stage and lactation. High exposure to folic acid can lead to saturation or inhibition of the dihydrofolate reductase enzyme, accumulating unmetabolized folic acid and decreasing the availability of methyl tetrahydrofolate (MTHF), which is the biologically active form of folate [33,38]. Studies have reported that high intakes of folic acid in early pregnancy are associated with the appearance of unmetabolized folic acid in the maternal circulation, suggesting that high dosages may be supraphysiological [38]. The higher concentrations may affect the offspring’s early development and postnatal growth (according to observed methylation patterns) [33]. In addition to the high doses, it is possible that the observed high folate concentrations were also related to the high prevalence of the methylenetetrahydrofolate reductase (MTHFR) polymorphism (38.4%), which has been described in the Mexican population [39].

Folic acid supplementation is a generalized nutrition intervention in prenatal care due to its positive effect on reducing the risk of neural tube defects (NTDs) and improving fetal growth [15]. Considering the recommendations of clinical guidelines for women with a higher risk of NTDs and/or folate deficiency, in clinical practice, it is expected to supplement women with metabolic disorders (obesity, diabetes, chronic kidney disease, bariatric surgery, liver disease) or those taking specific medications (metformin, antiepileptic) with very high doses of folic acid (4–5 mg/d). The recent guidelines for folic acid supplementation of the Society of Obstetrics and Gynecology of Canada establish that a high dose of 4–5 mg/d should only be recommended to high-risk women, which includes those with a personal history of NTD, a first-degree relative with NTD, or a previous pregnancy with NTD. The high doses should be given only from preconception to 12 weeks of gestation. Afterward, a lower dose supplementation scheme should be implemented (400 μg/d). Ideally, supplementation should be recommended as part of a multivitamin, with iron and 2.6 μg/d of vitamin B12 (100% of recommended intake) [14]. Supplementation with MTHF may be recommended because it does not require metabolization, specifically in persons with the MTHFR polymorphism [40], but more evidence is still needed.

Evidence from animal studies has shown that early excessive folate maternal exposure may be associated with altered DNA methylation patterns, disrupted embryonic and brain development, lower offspring body weight, and impaired offspring metabolic programming. However, more studies are needed to fully comprehend the potential long-term effects of high-dose folate supplementation [33,41,42]. These adverse effects may be mitigated by concurrent B12 supplementation [36]. In our study, vitamin B12 supplementation doses were also relatively high (median: 4.0; IQR: 2–12 μg/d). The latest National Health and Nutrition Survey in Mexico (ENSANUT) reported no folate deficiencies in adult women but low levels of B12 in 34% of them, which is similar to the prevalence of B12 we observed in the third trimester (32.2%) and aligns with findings from other studies [11,26,40,42].

It is important to emphasize that, in this study, we observed that high serum folate and low total B12 levels may coexist (serum and total). A high folate/low B12 ratio was present in 5% of women in the first trimester and increased significantly to 18.9% in the third trimester. This ratio has been associated with a higher risk of adverse outcomes, such as an increased risk of GDM, possible alteration in DNA methylation patterns, increased risk of LBW, and preterm delivery. Also, it may exacerbate increased adiposity and insulin resistance in children [16,17,25,43]. We found no association between a high folate/low B12 ratio and neonatal nutritional status. More research is needed to describe the possible effects of this high-risk ratio on maternal and infant outcomes. In addition, high folate concentrations may exacerbate a vitamin B12 deficiency in pregnancy [44,45]. B12 deficiency may lead to the development of metabolic diseases later in life. Studies in rats have shown that maternal B12 deficiency exhibited obesity and impaired lipid metabolism in their offspring [33].

Interestingly, homocysteine remained stable in the women in our study. In a review by Thakur P, it was described that Thcy levels fluctuate throughout pregnancy, decreasing as it progresses in normal pregnancy [46]. Thcy is a marker of altered one-carbon metabolism, rising in the presence of low folate or B12 due to decreased Thcy to methionine recycling. Nevertheless, a previous study found that high folic acid supplementation was not associated with lower Thcy concentrations [47].

Higher concentrations of Thcy have significantly been associated with altered neonatal nutritional status, predicting LBW, lower length, height, and head circumference [8,16,29,47,48]. Foremost, high maternal Thcy has been linked to placenta-mediated problems, which cause intrauterine growth restriction (IUGR), among other pregnancy complications, such as hypertensive disorders of pregnancy, abruption, and miscarriage, as a consequence of placental vasculopathy. However, the mechanisms remain unknown [46,49]. In line with this, we found a higher risk of stunting in women with higher levels of Thcy in the first trimester. Stunting at birth is a chronic representation of malnutrition and represents an abnormal growth pattern in utero. It has been associated with a higher risk of neonatal mortality. It is an important predictor for short- and long-term health and the risk of developing chronic diseases in adulthood [50]. We also observed a trend of association between higher maternal Thcy concentrations and lower birth weight, though it was not statistically significant. No folate deficiency was found in this study, which may explain why no elevated maternal Thcy was observed. More evidence is needed to clarify if the exacerbated B12 deficiency and continued high folic acid dose intake may cause a rise in Thcy levels over time.

B12 and folate are known to influence children’s linear growth potential. In this study, we did not find an association between this maternal B-vitamin concentration and the risk of stunting. However, a similar relationship has been described in children. In a Brazilian study, where they studied the association between high serum folate and low total B12 concentrations in children 6 to 59 months old and its effect on stunting, they found that children with normal/deficient folate concentrations and B12 deficiency had a higher risk of stunting than those with normal/deficient folate concentrations and normal B12 values (OR: 1.58; 95% CI: 1.02, 2.43), while the association between normal/high folate concentrations and B12 deficiency was not statistically significant (OR: 1.58; 95% CI: 1.02, 2.43) [51].

Previous studies have reported that maternal obesity/overweight is associated with a higher risk of vitamin B12 deficiency and lower folate levels [45,52]. One proposed mechanism for this association is the “folate trap”, where a decreased B12 intake or absorption leads to an accumulation of 5-MTHF that can exacerbate B12 deficiency [44,53]. This mechanism is considered a physiological response to a methyl donor group deficiency so that methionine can be saved for vital methylation reactions in the nervous system [44,53,54]. According to the most recent ENSANUT, 41.0% of Mexican adult women have obesity, and 35.8% are classified as overweight [55,56]. We did not find any association between maternal obesity and folate and B12 status, probably related to intensive supplementation prescribed to this group of women. We only observed a trend towards a lower concentration of active B12 in women with overweight and obesity. BMIpreg influenced no other marker.

This study presents some strengths, such as its prospective cohort design, where follow-up started from early pregnancy until birth (until this point), and the thorough evaluation of nutritional status, a facet often overlooked in birth studies, particularly regarding fat mass (FM). Additionally, including different vitamin B measurements at various time points during pregnancy allowed a more complete assessment of the maternal B vitamin status and how it can affect the offspring’s nutritional status. Our study also presents different limitations. The small sample size could decrease the power of the associations. The observational design does not allow us to establish causal associations. Also, our results may be influenced by genetic polymorphisms, which could differ by ethnicity, potentially affecting the results. Future steps should focus on expanding the sample size, incorporating genetic markers, and improving the evaluation of low B12 and high folate pattern (risk profile) during pregnancy. This may include using biomarkers such as methylmalonic acid, which could be more sensitive in detecting functional B12 deficiency. The evidence regarding cut-off points for defining folate deficiency and excess and vitamin B12 deficiency during pregnancy remains limited and lacks universal consensus. While some thresholds have been established to prevent severe conditions like megaloblastic anemia, there is no agreement on optimal folate and B12 levels for broader health outcomes, including neurodevelopment and metabolic programming. The absence of specific guidelines for vulnerable populations, such as pregnant women with unique physiological demands, combined with the variability introduced by genetic factors (e.g., MTHFR polymorphisms) and dietary patterns, underscores the need for more comprehensive research to refine recommendations and define optimal ranges for maternal and fetal health [57,58,59,60,61].

Further research is warranted to explore the long-term effects of maternal folate and B12 status on offspring metabolic health. Intervention studies are necessary to evaluate varying doses of folate, B12, and other B-complex vitamins in women with diverse baseline metabolism, nutrition profiles, and perinatal risk factors. Additionally, it is important to emphasize the potential need for personalized supplementation strategies during pregnancy tailored to maternal nutritional status, genetic variations such as MTHFR polymorphisms, and trimester-specific requirements. Early detection of folate and B12 deficiency may allow healthcare providers to individualize supplementation to ensure adequate levels while avoiding potential imbalances that may affect fetal growth and metabolic programming.

## 5. Conclusions

Our research highlights the intricate relationship between B12, folate, and homocysteine levels and newborn nutritional status and the importance of maintaining a balance in these nutrients. Higher maternal folate concentrations were associated with higher WC and FM% at birth, higher B12 concentrations were associated with higher birthweight and WC at birth, and higher maternal Thcy was associated with a higher risk of stunting at birth. The findings also shed light on the prevalence of high folate levels in some pregnant women, potentially due to high supplementation doses.

## Figures and Tables

**Table 1:** Maternal B vitamins and homocysteine concentrations in the first and third trimester according to maternal sociodemographic and clinical characteristics.

		First Trimester			Third Trimester		
Maternal Characteristics (*n* = 90)	All (%, *n*)	Total Vitamin B12 (pg/mol)	Serum Folate (ng/mL)	Homocysteine (µmol/L)	Total Vitamin B12 (pg/mol)	Serum Folate (ng/mL)	Homocysteine (µmol/L)
All		402.02 ± 313.11	25.40 ± 9.51	5.25 ± 1.11	296.21 ± 209.57	22.04 ± 9.38	4.78 ± 1.17
**Maternal BMIpreg** Normal Ow/Ob	46.6% (42) 53.3% (48)	477.26 ± 419.75 331.19 ± 142.63	24.39 ± 8.42 26.29 ± 10.38	5.08 ± 0.98 5.43 ± 1.21	321.76 ± 240.87 272.39 ± 177.50	20.41 ± 7.27 23.45 ± 10.78	4.81 ± 1.08 4.74 ± 1.24
**Parity** Nulliparous Multiparous	56.6% (51) 43.3% (39)	400.88 ± 309.38 399.05 ± 322.80	26.63 ± 10.72 23.72 ± 7.36	5.24 ± 1.05 5.30 ± 1.21	287.50 ± 176.90 307.58 ± 247.90	21.37 ± 6.51 22.92 ± 12.21	4.89 ± 1.22 4.61 ± 1.09
**Education** Basic Middle Higher	21.1% (19) 32.2% (29) 46.6% (42)	386.94 ± 172.65 334.03 ± 164.94 452.97 ± 421.01	24.61 ± 8.09 22.72 ± 10.17 27.72 ± 10.17	5.05 ± 0.89 5.46 ± 1.14 5.22 ± 1.18	265.94 ± 118.35 244.20 ± 85.97 345.80 ± 281.57	20.54 ± 6.65 19.20 ± 6.78 * 24.68 ± 11.25 *	4.81 ± 1.30 5.02 ± 1.09 4.57 ± 1.16
**Socioeconomic status** Low Medium/high	61.1% (55) 38.9% (35)	391.54 ± 322.43 421.17 ± 303.31	25.43 ± 9.34 25.70 ± 9.81	5.38 ± 1.22 5.02 ± 0.82	275.35 ± 177.18 335-76 ± 253.60	22.22 ± 7.13 22.21 ± 12.18	4.10 ± 1.26 4.68 ± 1.00

Mean ± SD is presented. U-Mann–Whitney test for normally distributed data/Kruskal–Wallis test for non-normally distributed data. * *p* < 0.05.

**Table 2:** Maternal B vitamin concentrations during pregnancy.

	First Trimester	Third Trimester	*p*-Value ^a^
Serum Folate (ng/mL)	25.40 ± 9.57	22.04 ± 9.38	0.037
RBC Folate (ng/mL)	666.32 ± 263.34	728.58 ± 369.86	<0.001
Total Vitamin B12 (pg/mol)	402.02 ± 313.11	296.21 ± 209.57	<0.001
Active Vitamin B12 (pmol/L)	76.30 ± 35.10	72.99 ± 32.48	<0.001
Homocysteine (µmol/L)	5.26 ± 1.11	4.78 ± 1.17	<0.001

^a^ Paired Student’s *t*-test.

**Table 3:** Maternal B vitamins and homocysteine concentrations in the first trimester according to neonatal characteristics.

Newborn Characteristics (*n* = 90)	All (%, *n*)	First Trimester				
		Total Vitamin B12 (pg/mol)	Active Vitamin B12 (pmol/L)	RBC Folate (ng/mL)	Serum Folate (ng/mL)	Homocysteine (µmol/L)
All		402.02 ± 313.11	76.30 ± 35.10	666.32 ± 263.34	25.40 ± 9.57	5.26 ± 1.11
**Sex** Male Female	43.3% (39) 56.7% (51)	458.29 ± 360.89 * 358.73 ± 270.43 *	78.72 ± 34.59 73.19 ± 34.93	686.36 ± 278.12 651.37 ± 253.51	26.54 ± 10.86 24.57 ± 8.40	5.10 ± 1.19 5.39 ± 1.04
**Resolution** Vaginal birth Cesarean section	44.4% (40) 55.6% (50)	390.24 ± 349.63 408.56 ± 281.93	73.29 ± 35.17 77.39 ± 34.77	646.57 ± 234.96 682.42 ± 285.80	23.86 ± 10.51 26.69 ± 8.49	5.20 ± 1.25 5.32 ± 1.00
**Gestational age** Preterm Term	8.9% (8) 91.1% (82)	310.57 ± 96.89 407.76 ± 324.24	60.12 ± 25.15 76.82 ± 35.19	669.85 ± 335.19 666.01 ± 258.86	20.35 ± 4.89 * 25.83 ± 9.70 *	6.04 ± 0.89 * 5.20 ± 1.11 *
**Stunting** Yes No	13.2% (12) 86.8% (78)	301.75 ± 111.12 416.24 ± 336.02	68.07 ± 31.17 76.17 ± 35.58	673.08 ± 265.69 661.72 ± 263.90	20.15 ± 5.30 ** 26.31 ± 9.89 **	5.21 ± 1.11 ** 5.17 ± 1.05 **
**Low birth weight** Yes No	13.2% (12) 86.8% (78)	301.54 ± 141.71 414.02 ± 328.51	48.13 ± 26.77 ** 79.74 ± 33.98 **	640.54 ± 231.17 669.94 ± 268.70	24.79 ± 7.27 25.50 ± 9.85	5.61 ± 1.42 5.21 ± 1.06
**BMI for age** Wasting Normal Ow risk	2.2% (2) 93.3% (84) 4.4% (4)	282.50 ± 104.94 408.66 ± 326.39 293.00 ± 67.60	34.00 ± 15.83 76.21 ± 34.90 72.22 ± 36.00	363.50 ± 51.61 672.16 ± 265.60 633.50 ± 178.62	23.75 ± 7.14 25.55 ± 9.86 24.72 ± 5.69	5.73 ± 2.65 5.26 ± 1.11 5.45 ± 0.70
**Small for gestational age** Yes No	15.5% (14) 84.5% (76)	430.90 ± 526.59 395.78 ± 275.58	68.47 ± 39.90 76.61 ± 33.99	626.58 ± 232.71 672.50 ± 268.63	24.11 ± 8.40 25.60 ± 9.71	5.66 ± 1.07 5.20 ± 1.11

Mean ± SD is presented. U-Mann–Whitney test for normally distributed data/Kruskal–Wallis test for non-normally distributed data. * *p* < 0.05. ** *p* < 0.01. BMI: Body mass index.

**Table 4:** Maternal B vitamins and homocysteine concentrations in the third trimester according to neonatal characteristics.

Newborn Characteristics (*n* = 90)	All (%, *n*)	Third Trimester				
		Total Vitamin B12 (pg/mol)	Active Vitamin B12 (pmol/L)	RBC Folate (ng/mL)	Serum Folate (ng/mL)	Homocysteine (µmol/L)
All		296.21 ± 209.57	72.99 ± 32.48	728.58 ± 369.86	22.04 ± 9.38	4.78 ± 1.17
**Sex** Male Female	43.3% (39) 56.7% (51)	317.923 ± 197.99 282.43 ± 217.702	81.26 ± 30.08 * 69.15 ± 33.71 *	748.02 ± 373.00 726.74 ± 375.78	21.33 ± 7.89 22.48 ± 10.48	4.76 ± 1.2 4.78 ± 1.15
**Resolution** Vaginal birth Cesarean section	44.4% (40) 55.6% (50)	274.25 ± 199.13 316.66 ± 216.69	70.46 ± 34.28 77.54 ± 31.13	716.525 ± 340.73 752.020 ± 399.505	20.366 ± 8.252 23.317 ± 10.188	4.79 ± 1.20 4.75 ± 1.15
**Gestational age** Preterm Term	8.9% (8) 91.1% (82)	230.37 ± 83.02 304.39 ± 216.56	62.65 ± 23.53 75.54 ± 33.21	857.75 ± 556.00 724.04 ± 362.18	19.83 ± 7.02 22.20 ± 9.64	5.43 ± 1.25 * 4.70 ± 1.14 *
**Stunting** Yes No	13.2% (12) 86.8% (78)	221.50 ± 78.14 307.46 ± 224.02	65.87 ± 20.39 73.65 ± 34.27	751.08 ± 450.20 724.54 ± 361.61	18.72 ± 4.88 22.52 ± 9.95	5.21 ± 1.11 4.73 ± 1.17
**Low birth weight** Yes No	13.2% (12) 86.8% (78)	210.00 ± 78.76 * 310.37 ± 220.03 *	51.19 ± 25.89 ** 77.39 ± 32.36 **	749.45 ± 377.18 612.81 ± 334.36	18.14 ± 4.48 22.52 ± 9.89	4.66 ± 0.99 5.51 ± 1.95
**BMI for age** Wasting Normal Ow risk	2.2% (2) 93.3% (84) 4.4% (4)	210.55 ± 75.66 304.09 ± 216.76 261.23 ± 110.50	47.95 ± 22.55 72.43 ± 32.68 78.11 ± 32.71	249.50 ± 53.03 737.16 ± 375.88 573.00 ± 294.84	14.12 ± 8.88 21.99 ± 9.60 23.08 ± 7.54	6.38 ± 2.12 4.76 ± 1.14 4.51 ± 1.02
**Small for gestational age** Yes No	15.5% (14) 84.5% (76)	210.26 ± 66.82 ** 329.72 ± 234.47 **	75.37 ± 32.40 68.59 ± 34.31	615.69 ± 295.60 726.65 ± 382.05	21.94 ± 9.65 21.99 ± 9.46	4.66 ± 1.07 5.43 ± 1.05

Mean ± SD is presented. U-Mann–Whitney test for normally distributed data/Kruskal–Wallis test for non-normally distributed data * *p* < 0.05. ** *p* < 0.01. BMI: Body mass index.

**Table 5:** Multivariate models: first-trimester B vitamins, homocysteine, and newborn nutritional status.

First Trimester						
Variable	B	Standard Error	*p*	95%CI		R2
				Lower	Upper	
**Birth Weight**			0.001			0.342
*Active B12*	0.002	0.001	0.021	0.000	0.004	
Gestational age	−0.646	0.12	<0.001	−0.884	−0.407	
**Waist circumference**			<0.001			0.162
*Active B12*	0.012	0.006	0.004	0.001	0.024	
Gestational age	−2.487	0.763	0.002	−4.007	−0.967	
**Waist circumference**			<0.001			0.173
*Total B12*	0.002	0.001	0.024	0	0.003	
Gestational age	−2.551	0.757	0.001	−4.059	−1.044	

Multiple linear regression. Models were adjusted by multivitamin supplementation, maternal pregestational body mass index, first-trimester gestational weight gain, gestational age at birth, and newborn sex. The primary study variables are presented in italics. Only statistically significant associations are presented (95% CI: <0 or >0, *p* < 0.05).

**Table 6:** Multivariate models: third-trimester B vitamins, homocysteine, and newborn nutritional status.

Third Trimester						
Variable	B	Standard Error	*p*	95% CI		R2
				Lower	Upper	
**Waist circumference**			<0.001			0.174
*Active B12*	0.016	0.006	0.011	0.004	0.028	
Gestational age	−2.212	0.75	0.004	−3.706	−0.717	
GWG 3T	0.103	0.046	0.027	0.012	0.194	
**Waist circumference**			<0.001			0.244
*Total B12*	0.003	0.001	0.007	0.001	0.005	
Gestational age	−2.172	0.747	0.005	−3.659	−0.685	
GWG 3T	0.121	0.046	0.01	0.03	0.213	
**Waist circumference**			<0.001			0.15
*Serum folate*	0.048	0.023	0.04	0.002	0.093	
Gestational age	−2.309	0.759	0.003	−3.82	−0.797	
GWG 3T	0.103	0.046	0.03	0.01	0.195	
Fat Mass (%) **			<0.001			0.245
*Serum folate*	0.127	0.047	0.009	0.033	0.220	
Sex	−2.304	0.972	0.021	−4.247	−0.361	
GWG 3T	0.289	0.11	0.01	0.07	0.509	

Multiple linear regression. Models were adjusted by multivitamin supplementation, maternal pregestational body mass index, third-trimester gestational weight gain, gestational age at birth, and newborn sex. Only statistically significant associations are presented (95% CI: <0 or >0, *p* < 0.05). The primary study variables are presented in italics. ** Subgroup of newborns with an air displacement plethysmography device measurements. 3T: Third trimester. GWG: Gestational weight gain.

**Table 7:** Association between maternal B vitamin and homocysteine status in the first and third trimesters and the risk of low birth weight and stunting.

Variable	*p*	Odds Ratio	95%CI	
			Lower	Upper
**Low birth weight**				
Active B12 1T	0.007	0.959	0.93	0.989
Active B12 3T	0.026	0.967	0.939	0.996
**Stunting**				
Thcy 1T	0.005	2.320	1.172	4.590

Multiple logistic regression models. Models were adjusted by multivitamin supplementation, maternal pregestational body mass index, first- or third-trimester gestational weight gain, gestational age at birth, and newborn sex. Only statistically significant associations are presented (95% CI: <1.0 or >1.0, *p* < 0.05). 1T: First trimester. 3T: Third trimester. Thcy: Homocysteine.

## Data Availability

The data presented in this study are available on request from the corresponding author. The data are not publicly available due to ethical reasons in accordance with the consent provided by participants on the use of confidential data.
